# Accelerating Effects of Poloxamer and Its Structural Analogs on the Crystallization of Nitrendipine Polymorphs

**DOI:** 10.3390/ph18071000

**Published:** 2025-07-03

**Authors:** Jie Zhang, Qiusheng Yang, Meixia Xu, Xinqiang Tan, Xucong Peng, Ziqing Yang, Kang Li, Jia Yang, Jie Chen, Xuan Xun, Saijun Xiao, Lingjie Zhou, Minzhuo Liu, Zhihong Zeng

**Affiliations:** 1 College of Biological and Chemical Engineering, Changsha University, Changsha 410022, China; zhangjie448215@163.com (J.Z.);; 2 Yantai Key Laboratory of Nanomedicine & Advanced Preparations, Yantai Institute of Materia Medica, Yantai 264000, China; 3 School of Pharmacy, Shenyang Pharmaceutical University, Shenyang 110016, China

**Keywords:** amorphous drug, poloxamer, crystallization, nitrendipine

## Abstract

**Background:** Surfactants can be added into polymer–amorphous drug systems to further enhance solubility. However, this may cause amorphous drugs to become physically unstable, and the inherent mechanism at play here is not fully understood. **Methods:** We explored the effects of poloxamer, a poly (ethylene oxide)-poly (propylene oxide)-poly (ethylene oxide) (PEO-PPO-PEO) triblock copolymer surfactant, and its segments on the nucleation and growth kinetics of amorphous nitrendipine (NTP) from the melt through polarized light microscopy. The effects of poloxamer and structural analogs on the melting point and glass transition temperature were also investigated using differential scanning calorimetry. **Results:** The poloxamer and its structural analogs enhanced nucleation and growth kinetics in supercooled liquid. Poloxamer and its structural analogs exhibited similar effects on the nucleation and growth kinetics of amorphous NTP, suggesting minimal dependence on structural variation. The overall crystallization rate of the NTP increased when increasing the poloxamer content and ultimately reached a maximum value; after that, the crystallization rates of NTP decreased when increasing the poloxamer content. **Conclusions:** Poloxamer and its structural analogs achieve similar effects on crystallization due to their comparable plasticizing effects. The nucleation and growth rates show different trends as a function of the poloxamer content. This effect is a result of both kinetic and thermodynamic factors. This study is relevant to understanding the impacts of the surfactant on the physical instability of amorphous drugs.

## 1. Introduction

Today, a growing array of drug substance candidates with poor aqueous solubility are being developed, with the application of molecular design and high-throughput screening [1]. Amorphous solids have received increasing attention due to their ability to enhance solubility and bioavailability for poorly water-soluble drugs. However, amorphous drugs with high free energy tend to recrystallize over time [2]. Amorphous drugs are commonly formulated with polymeric excipients to develop amorphous solid dispersions (ASD), a favored method for enhancing the physical stability of amorphous drugs [3]. The additives applied to ASD are of great importance and affect physical stability and bioavailability [4,5].

Surfactants are widely applied to enhance the dissolution rates and solubility of poorly soluble drugs [6]. Furthermore, the surfactants present in ASDs can serve as effective plasticizers, enhancing the melting process by reducing the melt viscosity, thereby facilitating the overall thermal processing [4]. The influence of surfactants on the crystallization process has been the subject of limited research, and the mechanism is still unclear [7,8]. Poloxamers are a class of semicrystalline poly (ethylene oxide)-poly (propylene oxide)-poly (ethylene oxide) (PEO-PPO-PEO) triblock copolymers containing PEO segments and PPO segments. Poloxamers have many grades according to the molecular weight and proportions of their two segments. Furthermore, they are commercially available under a range of brand names such as Pluronic^®^, and so, poloxamer P407 and Pluronic^®^ F-127 are essentially identical surfactants [9]. Poloxamers are frequently utilized as surfactants in ASDs due to their dual hydrophilic and hydrophobic properties [10,11]. Real et al. investigated the effects of applying a poloxamer on the solubility and dissolution of triclabendazole ASD [10,11]. They found that the drug solubility increased more than twenty times in the presence of a poloxamer. The degree of drug dissolution was also significantly increased by the poloxamer by helping to avoid drug precipitation [10,11]. In these studies, more attention is directed towards solubility and dissolution, rather than physical stability in a solid state. Poloxamers serving as polymeric carriers will crystallize in the final product; therefore, the poloxamer will exert a substantial impact on the amorphous drug. Hu and coworkers found that the poloxamer significantly affects the microstructures of drugs in crystalline SD [12,13]. Chen et al. also found that both PEG and PPG, as well as poloxamers, can significantly influence the microstructure during acetaminophen and bifonazole crystallization [14]. They also discovered that PEG, PPG, and poloxamers can significantly enhance the crystallization rates of drugs. This effect is primarily attributed to the substantial reduction in the glass transition temperature (*T*_g_) of the solid dispersions caused by these low-*T*_g_ polymers [14]. In recent research, Yao et al. found that surfactants including poloxamer could accelerate the crystallization of nifedipine by influencing the mobility of the drug [7]. In our latest study, we discovered that the poloxamer functioned as a plasticizer, substantially reducing the *T_g_* of clotrimazole. The poloxamer accelerated the crystal nucleation and growth of clotrimazole form 1 [8]. However, there is currently a lack of any quantitative understanding of how the structures of poloxamers and their various structural analogs affect nucleation and crystal growth rates.

In the present work, we used nitrendipine (NTP) as a model drug. NTP is a poorly water-soluble antihypertensive drug with low bioavailability, and is classically used for studying the crystallization of amorphous drugs [15,16,17,18,19,20]. In previous screening studies, it was found that the nucleation rate of NTP was moderate, which has encouraged further nucleation-related research [15]. Three polymorphs (Form I, II, and III) of NTP have been reported [16]. The additives selected in this study—poloxamer, polyethylene glycol (PEG), and polypropylene glycol (PPG)—have similar structures, but show differences in hydrophilicity and hydrophobicity. Regarding chemical structure, the study investigated the impact of poloxamer and its analogs on the nucleation and growth kinetics of amorphous NTP. In terms of additive content, the study also explored the nucleation and growth kinetics of amorphous drugs with varying poloxamer concentrations.

## 2. Results and Discussion

### 2.1. Crystallization of Pure NTP from the Melt

**Figure 1:**
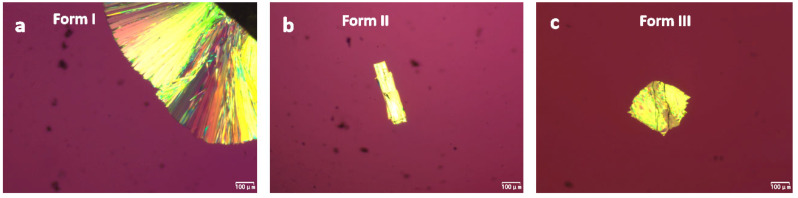
Photomicrographs of NTP crystals grown from the melt at 70 °C. Form I (**a**), Form II (**b**), and Form III (**c**).

As shown in Figure 1, three polymorphs of NTP were grown in supercooled liquid, which is consistent with a previous study [16]. Form I crystallized from the edge of the samples and formed compact spherulites. Forms II and III were prepared using a two-stage method, as referenced in [21,22,23]. Form II spontaneously nucleated between 30 and 70 °C and formed needle-like crystals in the first stage, after which, it formed aggregated crystals. Form III crystallized between 70 and 90 °C into coarse plates. Form II is the fast-nucleating polymorph; it formed within a few minutes of nucleation at 50 °C. Form III required several hours of nucleation at 70 °C. Forming a nucleus of Form I is generally more complicated; it is more random and is easier to achieve by seeding pre-existing Form I at the edge of the slide.

**Figure 2:**
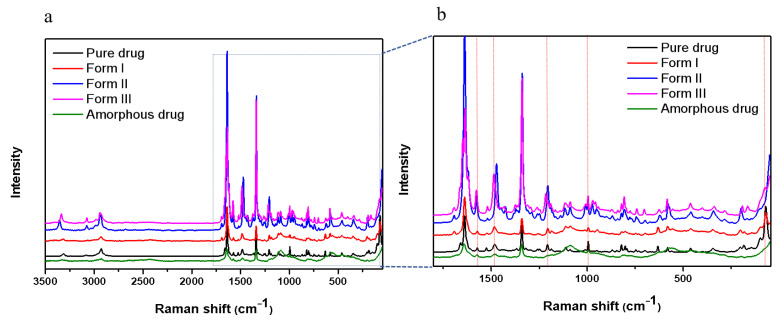
Raman spectra of NTP crystals grown from the melt between 50-3500 cm^−1^ (**a**) and magnified raman spectra between 50 and 1800 cm^−1^ (**b**).

**Figure 3:**
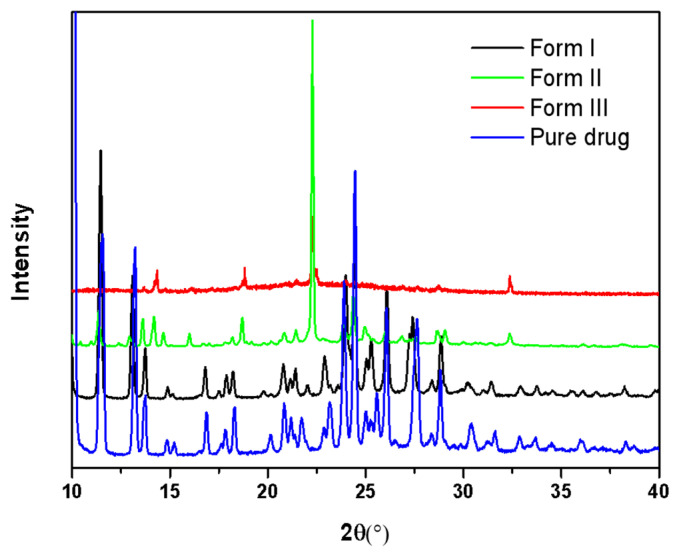
PXRD patterns of the NTP crystals grown from the melt.

The active pharmaceutical ingredient is Form I, as confirmed by PXRD. The PXRD result is in agreement with the documented structure reported in [16,24]. The structures of Form II and Form III are as yet unknown. We attempted to grow single crystals from the melt, but were unsuccessful, as either multiple polymorphs formed concurrently or a phase transition took place. Confocal Raman microscopy and PXRD (Figure 2 and Figure 3) were utilized to validate the polymorphism detected in the melt crystallization experiments. The PXRD results indicate that Form II contained a small amount of Form I. The PXRD pattern of Form III was found to be similar to that of Form II, likely due to the rapid conversion to Form II during the collection of the powder of Form III from a glass slide.

### 2.2. State of Mixing Between NTP and Additives

**Figure 4:**
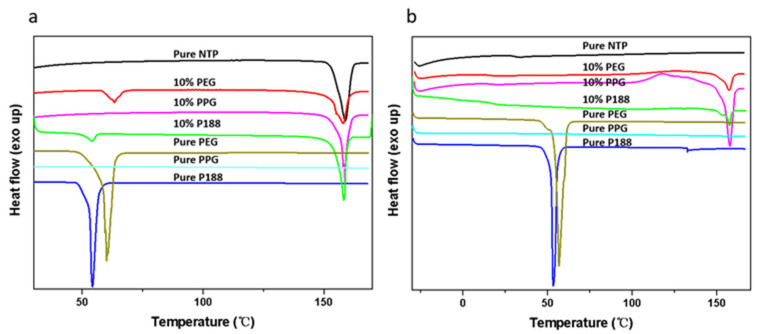
DSC traces of NTP–additive physical mixtures, pure NTP, and additives—first heating curve (**a**); second heating curve (**b**).

**Figure 5:**
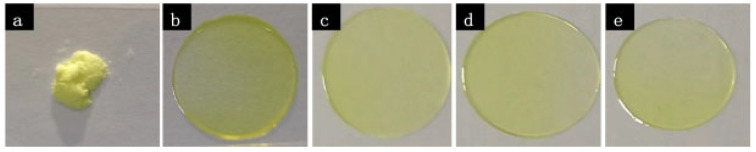
The appearance of pure drug and freshly prepared NTP/additive ASDs, NTP crystals (**a**), amorphous NTP (**b**), NTP containing 10% *w*/*w* PEG (**c**), NTP containing 10% *w*/*w* P188 (**d**), and NTP containing 10% *w*/*w* PPG (**e**).

Figure 4 illustrates the DSC curves for NTP (Form I) samples, each containing 10% *w*/*w* of various additives. It shows an endothermic peak of Form I at 158 °C, consistent with results from a previous study [16]. The *T_m_* values of Form II and Form III were defined on a hot stage. The melting point for Form II was approximately 130 °C, and that of Form III was around 120 °C, as detailed in the Supporting Information (Figures S1–S3). Both of these values are lower than the *T_m_* given for Form I. Figure 4a shows that the onset of NTP *T_m_* decreased when the sample was doped with additives. Figure 4b shows the second heating curve obtained after the melting point measurement. Compared to the pure drug, the solid dispersion system with the additive showed a melting peak of crystalline Form I, indicating that the additives had accelerating effects. The absence of crystalline Form II and Form III is due to the insufficient nucleation time. The *T_g_* of amorphous NTP was 32 °C. Only one *T_g_* could be observed when the amorphous solid dispersion was heated. Meanwhile, the freshly prepared NTP/additive ASDs were optically transparent, as depicted in Figure 5, and showed no birefringence when observed via polarized light microscopy. These observations indicate that NTP is miscible with the three additives. We should note that both P188 and PEG are semi-crystalline materials; meanwhile, they are prone to crystallization during the process of cooling after melting. Thus, melting peaks can be observed in both the initial and subsequent heating cycles, specifically within the temperature range of 50–60 °C. In the case of NTP containing 10% *w*/*w* P188 and PEG, the second heating process did not exhibit the melting peaks characteristic of P188 and PEG. This absence is attributed to the inhibitory effect of the drug, which aligns with findings from our previous studies [8,25,26].

### 2.3. Crystallizations of NTP Doped with Additives

**Figure 6:**
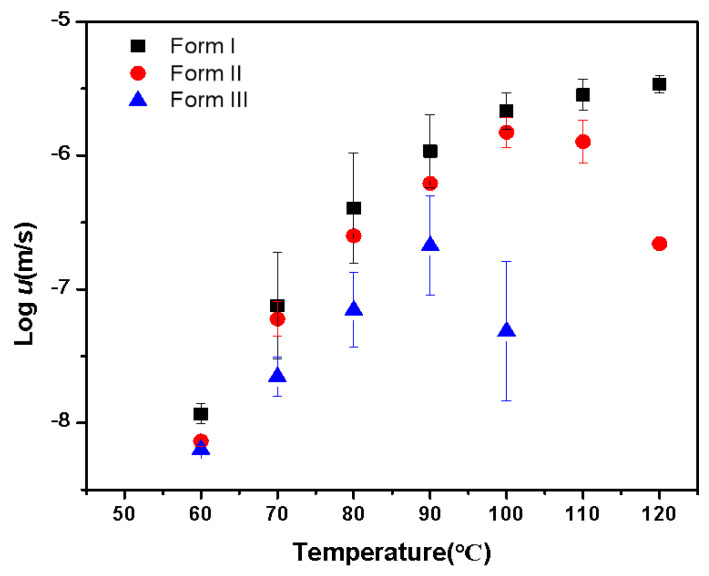
Crystal growth rate for amorphous NTP in relation to temperature.

Figure 6 illustrates the growth rates of NTP polymorphs as a function of temperature. Similar to the reported drugs, the growth rates show a familiar bell-shaped curve [27,28,29]. This outcome is a consequence of the interplay between thermodynamic and kinetic influences [27,28,29]. As temperatures decrease, molecular diffusivity is reduced, leading to slower crystallization rates. Conversely, from a thermodynamic perspective, increased supercooling (*T–T_m_*) provides a more vital driving force for crystallization. Consequently, the rates of crystal growth are expected to peak at a temperature that lies somewhere between *T_g_* and *T_m_*. The temperatures of maximum crystal growth rates for different polymorphs adopt the order Form I > Form II > Form III, which is correlated with the melting points of different polymorphs. The crystal growth rates also follow the order Form I > Form II > Form III.

**Figure 7:**
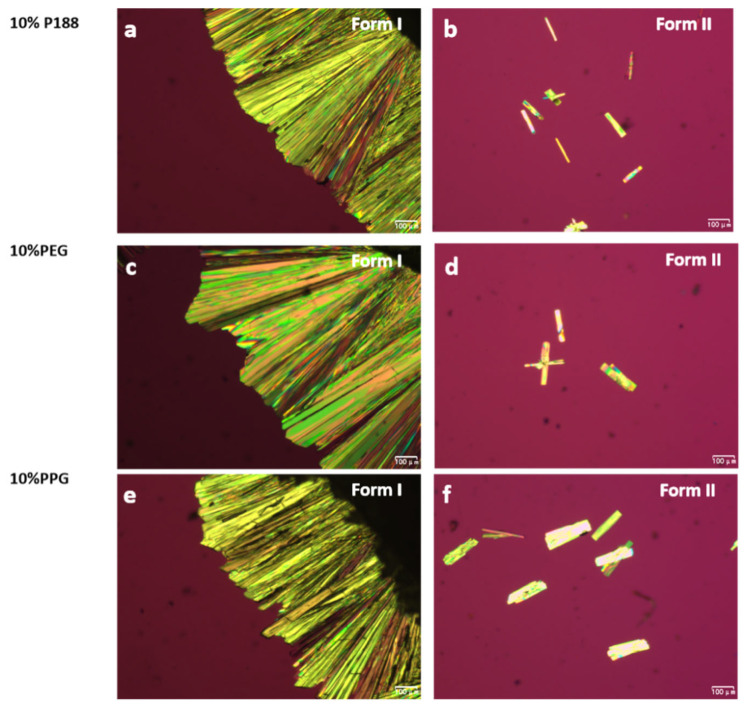
Crystal morphologies of NTP Form I and Form II grown in the presence of 10% *w*/*w* different additives, NTP containing 10% *w*/*w* P188 (**a**,**b**), NTP containing 10% *w*/*w* PEG (**c**,**d**), and NTP containing 10% *w*/*w* PPG (**e**,**f**).

Only Form I and II were observed in the presence of additives due to the relatively slow crystallization of Form III. Before the nucleation and growth of Form III, the liquid had already been consumed by Form I and Form II due to the accelerating effects of additives. Figure 7 shows the morphologies of NTP crystals grown with 10% *w*/*w* additives. Form I crystallized in the form of compact spherulites. Form II spontaneously nucleated and subsequently crystallized into needle-like crystals. The crystal morphologies of NTP closely resemble those of the pure drug, with the additives exerting minimal impact on the crystalline forms.

**Figure 8:**
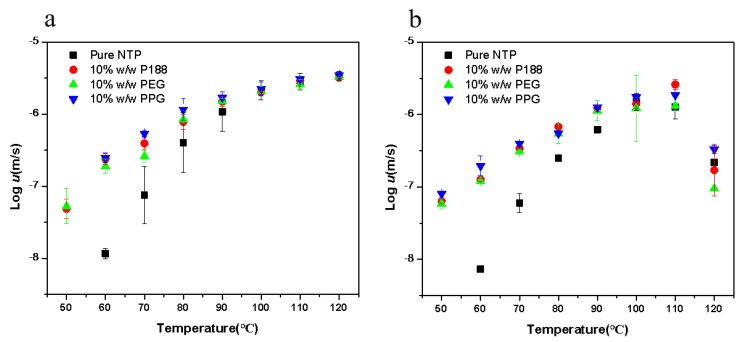
The kinetics of crystal growth for NTP Form I (**a**) and Form II (**b**) as a function of temperature.

Figure 8 presents the bulk crystal growth rates (denoted as *u*) for the polymorphs, in both the presence and absence of 10% *w*/*w* additives. When adding additives, the growth rates for NTP polymorphs were increased. The accelerating effects increase with cooling. Poloxamer and its segments showed similar accelerating effects, indicating that the structure of the additives is not the main factor influencing the growth rates. We also investigated the effects of the two types of poloxamer (P407 and P188) on the crystal growth of NTP Form I and Form II. The two types of poloxamer showed a similar effect on the two polymorphs (Figure S4). These findings suggest that alterations in the structure of poloxamer do not influence its ability to enhance the crystal growth rate of amorphous NTP. Yu and coworkers investigated the effects of different surfactants on the nucleation and growth rates of nifedipine [7]. They discovered that various surfactants exerted the same influence on the *T_g_* of nifedipine [7]. The effects of different surfactants were also similar independent of their structural details; thus, they attributed the similar effects of various surfactants on crystallization to their functions as mobility enhancers [7]. In this study, both poloxamer and its constituent segments exhibited comparable accelerating effects on the crystallization process, regardless of structural nuances. Figure 4 illustrates that the impacts of poloxamer and its segments on the glass transition temperature *T_g_* of NTP were consistent, despite their varying structural features and hydrophilic–lipophilic properties. While some studies on NTPs have shown that interactions between polymers and drugs can influence crystallization [15], interactions of poloxamer and its structural segments with the drug may also have occurred in this study, but they were likely not the primary factors affecting crystallization. It appears that the mobility of the drug is the pivotal factor dictating the crystal growth of NTP.

**Figure 9:**
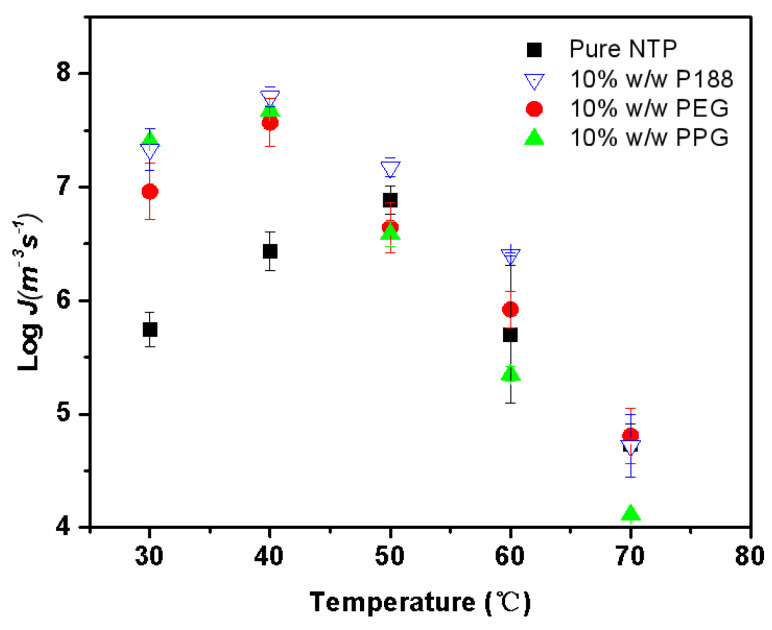
The kinetics of crystal nucleation for NTP Form II as a function of temperature.

Figure 9 illustrates the bulk crystal nucleation rates (denoted as *J*) for Form II with and without the inclusion of 10% *w*/*w* additives. In this section, we only investigated the nucleation rate of Form II due to its main crystal nucleation in the sample. We tried our best to determine the effect of the segment on the nucleation rate of Form I (similar to the data for Form II in Figure 9). However, determining the nucleation rate of Form I was challenging because Form II nucleates more readily, while Form I nucleates slowly, appears at higher temperatures, and exhibits greater randomness. The amorphous samples tend to be rapidly occupied by Form II, making it difficult to obtain nucleation data for Form I. The nucleation kinetics of Form II manifested a bell-shaped curve, and the temperature of the maximum nucleation rate was well below the maximum crystal growth rate. Similar phenomena are observed for other drugs, such as fluconazole [3], clotrimazole [30], acetaminophen [31], etc. This can be described by classical nucleation theory (CNT), as shown in Equations (1) and (2).

According to the CNT, the nucleation rate can be expressed as:

(1) $$J=k_{J}\exp\left(-W_{c}/kT\right)$$

where $W_{C}=\frac{16\pi }{3}\frac{\sigma^{3}}{\Delta G_{V}^{2}}$ is the thermodynamic barrier for forming a critical nucleus, σ is the interfacial energy between the crystal nucleus and liquid, ∆*G_v_* is the energy difference between the crystal and the liquid, and *k_J_* is a kinetic factor that can be represented as a function of the dynamics of the liquid phase.

The difference in ∆*G_v_* can be calculated using the Hoffman equation:

(2) $$\Delta G_{V}=\frac{\Delta H_{f}(T_{m}-T)T}{T_{m}^{2}}$$

where ∆*H_f_* is the heat of fusion and *T_m_* is the melting point.

The poloxamer and its segments show similar accelerating effects on nucleation rates. For neat amorphous NTP, nucleation of the Form II reached a maximum value at 50 °C. Miyazaki et al. studied the nucleation rates of NTP enantiomers, and the results show that the maximum nucleation rates were also near 50 °C there [15]. Nucleation of Form II reached its peak at 40 °C upon the addition of additives. Within the thermodynamic domain, the additive exerted a minimal influence on the nucleation rate. However, in the kinetic region, the nucleation rate increased by at least an order of magnitude, resulting in a shift in the maximum nucleation rate to an earlier time point. From Figure 4, it can be observed that the addition of 10% *w*/*w* of the three additives had a minor impact on the *T_m_*, but a significant effect on the *T_g_*. This disparity highlights the differences in the additives’ effects on kinetics and thermodynamics according to CNT, ultimately leading to their varying impacts in different regions. In the kinetic region, the three additives showed the same accelerating effect on the nucleation rate, which is consistent with their impact on the *T_g_*. Our results are similar to those of previous reports [7]. Yu and colleagues examined the impacts of various surfactants on the nucleation of nifedipine [7]. Despite the structural diversity and varying HLB values among these surfactants, their primary roles appear to be as mobility enhancers [7]. These results diverge from those derived with conventional polymers, which typically exhibit inhibitory effects on crystallization. These polymers frequently exert significant effects on the crystallization process, attributable to a combination of factors such as intermolecular interactions, antiplasticization effects, segmental mobility, etc. [4,15,16,17,18,19,20].

### 2.4. Different Effects on Nucleation and Growth Rates with Increasing Poloxamer Content

**Figure 10:**
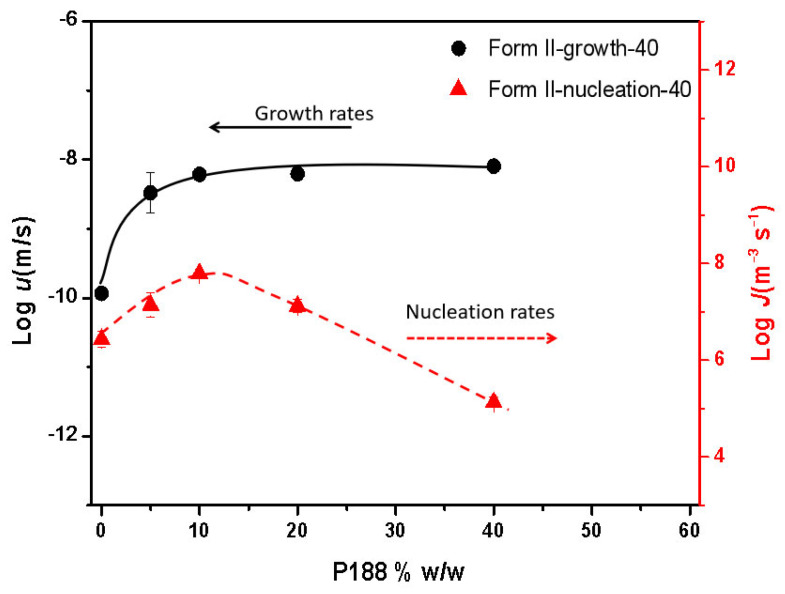
Crystal nucleation and growth kinetics of amorphous NTP as a function of P188 content at 40 °C.

Figure 10 illustrates the growth kinetics of NTP crystal Form II containing different amounts of P188 at 40 °C. The crystal growth rates plateaued when the P188 loading reached 10% *w*/*w* at temperatures of 40 °C. This phenomenon, as previously discussed in our research, is affected by the local phase separation that occurs at the crystal growth front [8,25,26]. The crystallization rate remains constant due to the enrichment at the crystallization front, where the concentration of additives is maintained at a consistent specific level [8,25,26]. Regarding nucleation, Figure 10 illustrates that the nucleation rates of NTP crystal Form II increased as the P188 content rose, particularly at low concentrations of P188. It has been documented that additives exert comparable influences on both crystal nucleation and growth, attributable to their analogous effects on the molecular mobility that is integral to these processes. Consequently, it can be predicted that nucleation will follow a similar trend [3,32]. In this study, a similar phenomenon was observed at low P188 concentrations. No plateaus were observed with further increases in P188 content; the nucleation rates peaked and then subsequently declined. It was found that the maximum nucleation rate occurred at 40 °C and a P188 concentration of 10%, while at the same temperature, the maximum nucleation rate was achieved at a P188 concentration of 5% *w*/*w*. Comparing nucleation rates and growth rates, we found that nucleation rates were more susceptible to the influence of P188 content. For crystal growth, there was an enriched layer on the growth front, and the situation for drug molecules remained relatively stable; thus, the growth rates reached a plateau, while for nucleation, with increasing additive contents, nucleation decreased. According to classical nucleation theory, when clusters grow to larger than critical sizes as a result of a fluctuation, nuclei will form [33,34]. The number of molecules constituting the critical nuclei usually falls in the range of 10−1000 [33,34]. The presence of doped additives in the system may affect critical nuclei’s formation, thus impacting nucleation rates.

**Figure 11:**
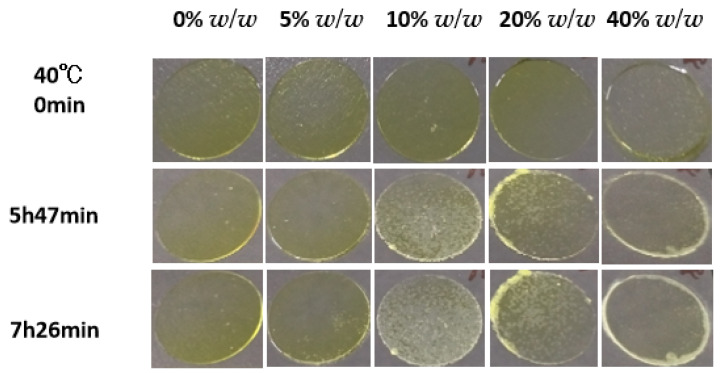
Crystal growth kinetics of amorphous NTP in the presence of P188 as a function of content at 40 °C.

Figure 11 shows the overall crystallization of amorphous NTP in the presence of P188 as a function of content at 40 °C. Consistent with the results shown in Figure 10, the maximum overall crystallization nucleation rate at 40 °C was reached for 10% *w*/*w* P188, taking into account the impacts of nucleation and crystal growth. Figure 11 shows the growth of a very large spherulite at 40 °C with 40% *w*/*w* P188 (rightmost column). In this scenario, the crystals that formed were of Form I. The nucleation of Form II was suppressed, allowing Form I to nucleate and grow at a faster rate than Form II. Concurrently, Form I crystals emerged at the edges of coverslips when increasing the P188 content—a phenomenon primarily attributed to rapid surface crystallization at these edges.

### 2.5. Effects of T_g_ and T_m_ on Crystallization Kinetics

**Figure 12:**
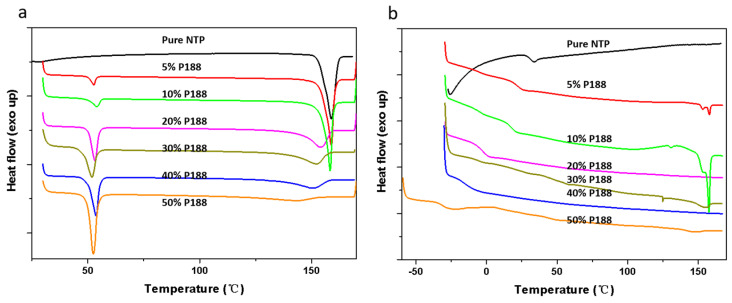
DSC traces of NTP-P188 mixtures (**a**) and ASD samples containing 0–50% *w*/*w* P188 (**b**).

**Figure 13:**
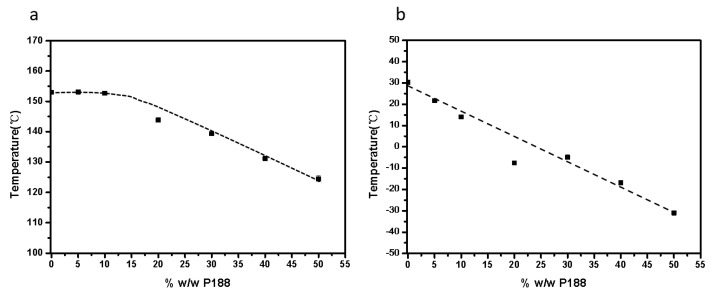
*T_m_* values (**a**) and *T_g_* values as a function of amorphous NTP samples containing 0–50% *w*/*w* P188 (**b**).

The influence of additives on drug crystallization has been extensively explored in prior research [4]. The impact of the *T_g_* is a crucial factor in the crystallization of amorphous drugs. Sato and colleagues studied the effects of a biocompatible polymer with a low *T_g_* on the crystallization of diverse organic compounds [35]. They assessed the influences of varying polymer concentrations on crystallization rates by normalizing these rates against the *T_g_*. Their findings reveal that the growth rates of compounds with different polymer concentrations, plotted against the temperature difference (*T*–*T_g_*), were comparable to those observed for the pure drug [35]. The low-*T_g_* additives increased the crystal growth rate by facilitating mass transport effects [35]. In this study, as depicted in Figure 12 and Figure 13, the *T_g_* values progressively decreased with the increasing content of P188. When comparing the crystal nucleation and growth kinetics (Figure 10) with the *T_g_* values, we see that the *T_g_* values progressively decreased with an increase in P188 content. In contrast, the nucleation and growth rates showed different trends. The decreased *T_g_* values represent evidence that P188 could enhance the mobility of the drug. It seems that additional factors, such as local phase separation, also play a role in influencing nucleation, as discussed in Section 3.4. *T_g_* values are thus not predictive indicators of changes in crystallization kinetics with P188 content.

The additives could significantly decrease the *T_m_* and heat fusion of the drug, regardless of whether the polymers have high or low *T_g_* [26,36]. In this work, we find that the additives have a relatively minor impact on *T_m_* and heat fusion when their content is below 10% *w*/*w* (as shown in Figure 12 and Figure 13). However, when the content of P188 exceeds 10% *w*/*w*, the *T_m_* and heat fusion undergo significant changes. According to Equations (1) and (2), the decreased *T_m_* and heat fusion could affect the ∆*G_v_* and thus influence nucleation rates.

Miyazaki and colleagues investigated the influence of polymer additives on the nucleation rates of NTP enantiomers, and found that stereoselective interactions affected the crystallization process [15]. While these polymers function as crystallization inhibitors due to their high *T_g_*, the crystallization process is still subject to a variety of influencing factors [15]. For additives with a low *T_g_*, when the system contains low-concentration additives, the nucleation and crystal growth rate are enhanced and show similar trends. In this stage, the structure is not a very important factor, and the two processes are governed by mobility. When the concentration of additives increases further, the crystal growth rates reach a plateau, while the nucleation rates decrease after reaching a maximum value. Regarding overall crystallization, crystallization is accelerated with increasing additive content when the content is low, and the accelerating effects decrease with further increases in the additive content. Both thermodynamic and kinetic factors likely influence the nucleation and crystal growth of amorphous nitrendipine as induced by poloxamer. At lower concentrations, the molecular mobility of poloxamer and its influence on the molecular mobility of the amorphous drug is dominant. However, as the concentration increases, the contributions of thermodynamic factors become more significant. Regarding this phenomenon and its implications for determining the optimal poloxamer concentration to balance solubility enhancement and physical stability, we can conclude that when incorporating poloxamer to enhance the solubility of ASDs, its concentration should be maintained at the minimum level necessary to achieve the desired solubility enhancement. Although further increasing the amount of poloxamer tends to decrease the crystallization rate, this effect is often significant only at very high concentrations of poloxamer. Given the dosage limitations, such high concentrations are impractical, and may actually hinder the development of ASDs. Whether the use of surfactants or plasticizers, such as water, yields similar conclusions is worthy of further investigation.

## 3. Materials and Methods

### 3.1. Materials

**Figure 14:**
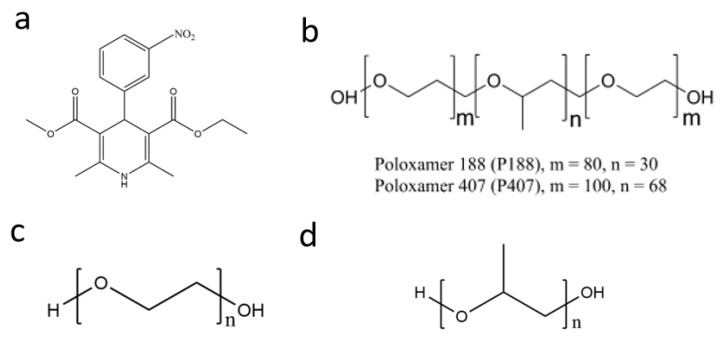
Structures of nitrendipine (**a**), poloxamer (**b**), poly(ethylene oxide) (**c**), and poly(propylene oxide) (**d**).

Nitrendipine was purchased from Bide Pharmatech Co., Ltd., Shanghai, China (purity > 98.0%, Form I, used in the form of a racemic mixture); PPG (Mn = 4000) was obtained from Bide Pharmatech Co., Ltd, Shanghai, China.; and PEG (Mn = 4000) and poloxamer 407 and 188 were obtained from BASF SE, Ludwigshafen, Germany (Figure 14).

### 3.2. Preparation of Drug/Additive Physical Mixtures and ASDs

Physical mixtures of drugs and additives, varying in additive content (0, 5, 10, 20, 30, 40 and 50% *w*/*w* of additive), were meticulously prepared using a mortar and pestle. The preparation of ASDs involved melting 2–5 mg of drug/additive mixtures at a temperature of 165 °C between two round glass coverslips for 1 min. This process was followed by rapid cooling to room temperature to achieve the desired amorphous state.

### 3.3. Polarized Light Microscopy (PLM)

The crystalline morphologies were examined by PLM (Guangzhou Micro-Shot Technology, Guangzhou, China), while temperature control during the process was meticulously managed using a hot stage (Beijing Shiji Kexin Scientific Instrument, Beijing, China) or oven.

### 3.4. Powder X-Ray Diffraction (PXRD)

PXRD analysis was conducted using a Bruker D8 Advance equipped with Cu Kα radiation, characterized by a wavelength of 1.542 Å. The samples were mounted on a zero-background silicon sample holder, and the scanning was performed over a 2θ range of 10° to 40° at 10°/min.

### 3.5. Raman Microscopy

Raman spectroscopy was carried out utilizing a confocal Raman Microscope (DXR, Thermo Fisher Scientific, Madison, WI, USA), which was equipped with a 780 nm laser source. The spectra were acquired using a 50× objective lens with a total exposure time of 20 s (1 s acquisition × 20).

### 3.6. Differential Scanning Calorimetry (DSC)

DSC was conducted using a Q2000 system (TA Instruments, New Castle, DE, USA). The analysis was performed under a 50 mL/min N_2_ purge. Approximately 3–5 mg of the powdered sample was accurately weighed and placed in an aluminum pan. To obtain the *T_m_* (melting temperature) values, the sample was initially heated to 170 °C at a rate of 10 °C per minute. After that, it was cooled down to −30 °C, and then subjected to a re-heating cycle to 170 °C at the same rate of 10 °C per minute to determine the *T_g_* values.

### 3.7. Crystal Growth Rates of NTP

Each sample, weighing 2–5 mg, was heated to 165 °C on the hot stage between two glass coverslips and then rapidly quenched to room temperature. The crystallization of polymorphs was initiated either spontaneously or through seeding, after which, the samples were set to a predetermined temperature for growth. The crystal morphology and size were observed and recorded using a PLM. The growth rates of the polymorphs were determined in triplicate to ensure the accuracy and reliability of the measurements. In this study, the growth rates we compared were all within the diffusion region.

### 3.8. Nucleation Rates of NTP

The nucleation rates were ascertained using a two-stage method, as referenced in [21,22,23]. Nuclei were successfully formed at the target temperature on the hot stage, and subsequently, the crystals grew to visible sizes upon heating to 100 °C. The crystal morphology and quantity were observed and recorded with a PLM. In the current study, samples weighing between 2 and 5 mg were melted beyond their respective melting points, then rapidly cooled to the target temperature and maintained there for varied durations to facilitate nucleation. As the nucleation time was extended, the number of crystals increased. Thus, it was feasible to statistically analyze the relationship between the numbers of crystals per unit volume as a function of time, which allowed us to subsequently ascertain the nucleation rate. The nucleation rates were measured by quantifying the number of nuclei formed per unit volume over a given period. When plotting the number of nuclei per unit volume against time, the slope of the curve represents the nucleation rate.

## 4. Conclusions

In this work, we evaluated the effects of poloxamer and its segments on the nucleation and growth kinetics of NTP. The poloxamer here functioned as a plasticizer, substantially reducing the *T_g_* of the NTP. The poloxamer and its structural analogs had similar impacts on the nucleation and growth processes of the amorphous drug, suggesting minimal dependence on structural variation. The nucleation and growth rates showed different trends with increasing poloxamer content. The effect of the poloxamer on the crystallization of NTP arises from thermodynamic and kinetic interactions. The overall crystallization rates of NTP increased when increasing the poloxamer content and reached a maximum value; after that, the crystallization rates of NTP decreased when increasing the poloxamer content. Investigating the effects of these three structurally similar additives on the crystallization and nucleation of NTP will aid in explaining the mechanism by which polymer additives influence the nucleation of amorphous drugs. This indicates that the concentration of poloxamer in ASDs should be meticulously determined, as the polaxamer’s impact on physical stability exhibits a non-linear relationship with the amount present.

## Data Availability

Data will be made available on request.

## Supplementary Materials

The following supporting information can be downloaded at: https://www.mdpi.com/article/10.3390/ph18071000/s1, Figure S1. Photomicrographs of NTP Form I at 159 °C at different times. Figure S2. Photomicrographs of NTP Form II at 130 °C at different times. Figure S3. Photomicrographs of NTP Form III at 120 °C at different times. Figure S4. Crystal growth kinetics for NTP Form I (a) and Form II (b) in the presence of 10% *w*/*w* P407 or P188 as a function of the temperature.
