# Supplementary material for: Accelerating Effects of Poloxamer and Its Structural Analogs on the Crystallization of Nitrendipine Polymorphs
Source: Pharmaceuticals (Basel). 2025 Jul 3;18(7):1000. doi: 10.3390/ph18071000 (PMC12298067; doi:10.3390/ph18071000)
Supplement: Supplementary file 1 [file pharmaceuticals-18-01000-s001.zip › pharmaceuticals-3664717-supplementary.pdf]

Accelerating Effects of Poloxamer and Its Structural Analogs on the Crystallization of Nitrendipine Polymorphs

Jie Zhang [1,†], Qiusheng Yang [1,†], Meixia Xu [2], Xinqiang Tan [3], Xucong Peng [1], Ziqing Yang [1], Kang Li [1], Jia Yang [1], Jie Chen [1], Xuan Xun [1], Saijun Xiao [1], Lingjie Zhou [1], Minzhuo Liu [1,*] and Zhihong Zeng [1,*]

[1] College of Biological and Chemical Engineering, Changsha University, Changsha 410022, China; zhangjie448215@163.com (J.Z.); yang_qiusheng@gibh.ac.cn (Q.Y.); pengxc1275929532@outlook.com (X.P.); y3296093276@163.com (Z.Y.); 15572133315@163.com (K.L.); 15773114421@163.com (J.Y.); 19169957260@163.com (J.C.); xunxuan18333623272@icould.com (X.X.); xsaijun@126.com (S.X.); qw3369703826@outlook.com (L.Z.)

[2] Yantai Key Laboratory of Nanomedicine & Advanced Preparations, Yantai Institute of Materia Medica, Yantai 264000, China; mxxu@yimm.ac.cn

[3] School of Pharmacy, Shenyang Pharmaceutical University, Shenyang 110016, China; t304594431@126.com

* Correspondence: z20200821@ccsu.edu.cn (M.L.); z20181201@ccsu.edu.cn (Z.Z.)

† Contributed equally to this work.

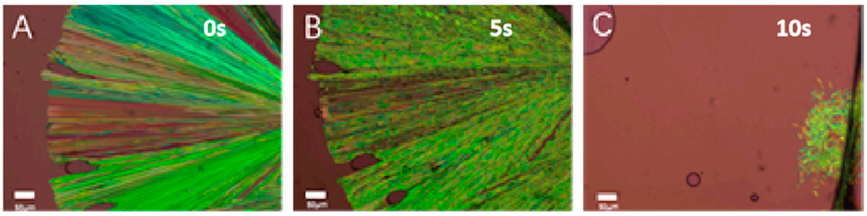

Figure S1. Photomicrographs of NTP Form I at 159 °C at different times.

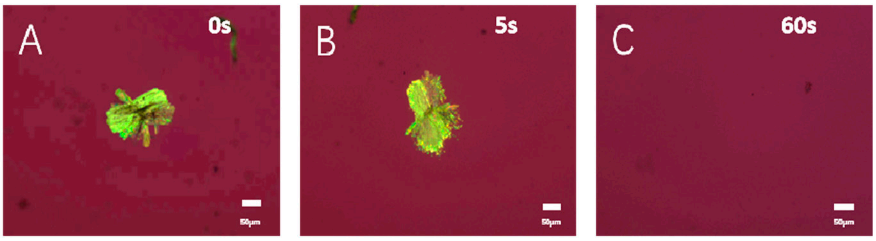

Figure S2. Photomicrographs of NTP Form II at 130 °C at different times.

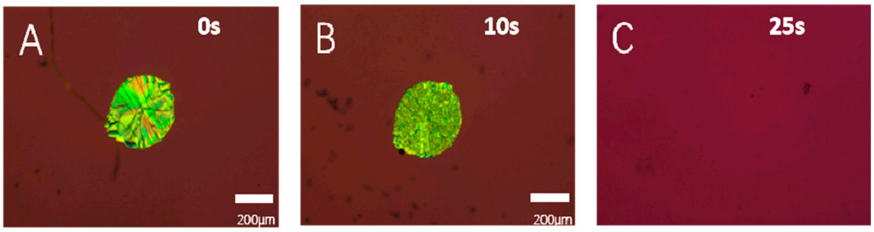

Figure S3. Photomicrographs of NTP Form III at 120 °C at different times.

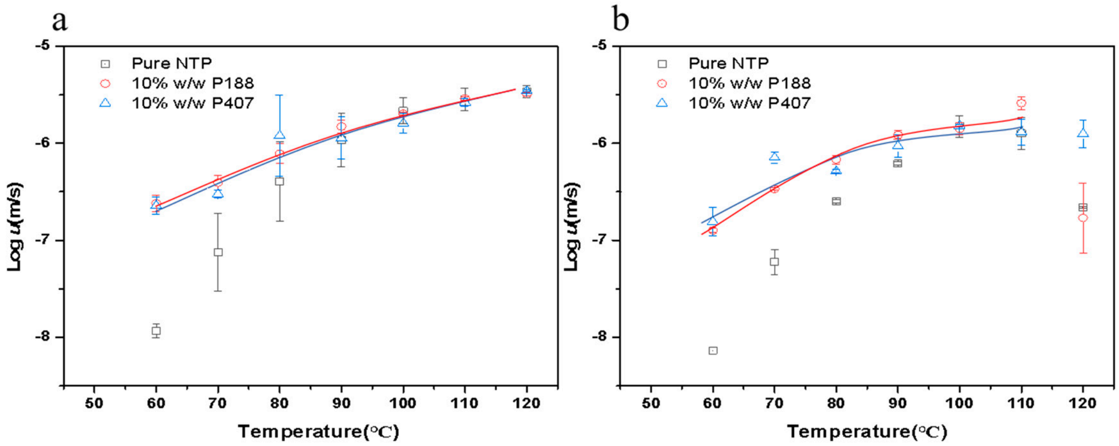

Figure S4. Crystal growth kinetics for NTP Form I (a) and Form II (b) in the presence of 10% w/w P407 or P188 as a function of the temperature.
